# The perplexing problem of persistently PCR-positive personnel

**DOI:** 10.1017/ice.2020.343

**Published:** 2020-07-20

**Authors:** David K. Henderson, David J. Weber, Hilary Babcock, Mary K. Hayden, Anurag Malani, Sharon B. Wright, A. Rekha Murthy, Judith Guzman-Cottrill, Sarah Haessler, Clare Rock, Trevor Van Schooneveld, Latania Logan, Corey Forde

**Affiliations:** 1 National Institutes of Health, Bethesda, MD; 2 University of North Carolina at Chapel Hill, Chapel Hill, NC; 3 Washington University School of Medicine, St. Louis, MO; 4 Rush University Medical Center, Chicago, IL; 5 St. Joseph Mercy Health System, Ann Arbor, MI; 6 Beth Israel Deaconess Medical Center, Boston, MA; 7 Cedars-Sinai Health System, Los Angeles, CA; 8 Oregon Health and Science University, Portland, OR; 9 Baystate Health, Springfield, MA; 10 Johns Hopkins University School of Medicine, Baltimore, MD; 11 University of Nebraska Medical Center, Omaha, NE; 12 Queen Elizabeth Hospital, Barbados

Early in the coronavirus disease 2019 (COVID-19) pandemic, the Centers for Disease Control and Prevention (CDC) published return-to-work criteria for healthcare personnel who had recovered from severe acute respiratory coronavirus virus 2 (SARS-CoV-2) infection. These criteria were most recently updated on April 3, 2020.^1^ The CDC has endorsed 2 different approaches to allow staff to return to work: a symptom or time-based strategy and a test-based strategy. Many institutions initially adopted the test-based strategy, in part because CDC initially recommended it as the preferred option (but no longer does so) and, in part, because it seemed the more definitive or conservative of the 2 CDC options.

As a result of using the test-based strategy, many institutions now have significant numbers of staff whose nasopharyngeal swabs remain RT-PCR positive for COVID-19, despite the fact that they have recovered from their episodes of illnesses and have been asymptomatic for weeks or, in some instances, months. These staff effectively remain in limbo; they feel well but have persistently positive PCR studies, and driven by the test-based strategy, they cannot return to the workplace until they have 2 negative COVID-19 PCR tests.

This phenomenon (persistently positive PCR tests for extended periods after recovery from COVID-19 infection) has now been well described in the literature, including a paper describing the first 12 COVID-19 cases in the United States,^2^ a detailed study of 9 cases from Germany^3^ and a recent paper from Manitoba.^4^ Similarly, Strong and Feldmann described a lack of clear linkage between PCR positivity and viral infectiousness during the Ebola epidemic.^5^ The Center for Infectious Diseases of the National Academy of Medicine of Singapore issued a position statement, including data demonstrating that, of 73 COVID-19 patients, virus was not cultivable in any of them after day 11 of their illnesses.^6^ Similarly, the Korean Centers for Disease Control evaluated 285 patients who were found to be PCR positive for COVID-19 8–82 days following recovery from a documented COVID-19 illness.^7^ They were able to attempt viral culture in 108 of these individuals and could not isolate virus from any of them.^7^ Each of the COVID-19 studies cited here performed viral culture in addition to PCR testing for viral RNA. In each of the studies, coronavirus RNA could be detected long after virus could no longer be cultured from upper respiratory samples. These data document that individuals who had illnesses not requiring hospitalization, who recovered from the disease and who remain persistently positive by PCR, no longer harbor cultivable virus 10 days after symptom onset. Notably, these studies have assessed relatively healthy populations who developed COVID-19 infection but were not severely ill. We do not yet know whether the 10-day cutoff is appropriate for immunocompromised healthcare personnel (HCP) who develop COVID-19, but it clearly does not apply for HCP who suffered severe disease (eg, with hospitalization and/or prolonged intubation).^8,9^ Such individuals will have to be managed on a case-by-case basis until more data become available.

In the Canadian study cited above, the authors retrospectively evaluated 90 previously identified positive samples by both PCR and viral culture in Vero-cells. They compared both the number of days from onset of symptoms to the day the test was performed as well as the cycle thresholds of PCR positivity to recovery of viable virus in tissue culture.^4^ In no instance were the investigators able to recover virus if >8 days had elapsed from the onset of symptoms, despite the persistence of positive PCR tests. In addition, if the cycle threshold for the sample was >24, they could not recover virus in tissue culture.^4^

Relying on a cycle threshold limit as a return-to-work criterion has some limitations. Cycle thresholds are not directly comparable from site to site or even from test to test. In addition to the number of copies of RNA in a given sample, cycle thresholds depend on a variety of factors, including the specific gene target(s) and the number of gene targets chosen for the assay, the platform used, variability in reagents used from site to site, and more. Optimally, to use the cycle threshold, an institution would validate the procedure in its own laboratory, demonstrating, as the study from Manitoba did, that samples above a certain cycle threshold did not contain cultivable virus. The challenge in doing such a validation is that you need a Biosafety Level 3 laboratory to do the tissue culture work, and many, if not most centers will not have access to those resources. Additionally, if any aspects of the PCR assay change, revalidation is appropriate. Another challenge in using cycle thresholds is that not all diagnostic nucleic acid amplification assays use RT-PCR, so cycle thresholds are not available, and some rapid instruments use PCR but do not yield cycle threshold values. Finally, regarding the standardization of testing, if the clinical laboratory community developed universal standards for SARS-CoV-2 similar to the World Health Organization’s standards for hepatitis B and C, a multicenter study of all currently manufactured SARS-CoV-2 nucleic acid amplification tests could be designed to correlate the cycle threshold values on each platform for patients who have positive and negative viral cultures. Clinical laboratories could then calibrate their assays using the universal standard, thus allowing laboratories to estimate virus viability from the cycle threshold with some certainty. To assure proficiency and reliability, the College of American Pathologists could develop proficiency panels for labs for quality assurance. This standardization scenario represents a desired future state; for the time being, we will have to rely on other strategies.

Conversely, relying on time from symptom development as a return-to-work criterion is also fraught with a few challenges. The onset of COVID-19 may be insidious and may be difficult for an individual provider to pin down. Recall bias may affect the provider’s ability to be precise about the onset of symptoms. As asymptomatic provider surveillance testing expands, protocols will have to be developed to manage asymptomatically infected individuals.

So how do we approach these issues, balancing the safety of patients and staff with the need to avoid having staff unnecessarily sidelined staff who could be contributing to an important pandemic response when we need them the most? Given the data cited here, the test-based strategy appears likely to delay HCP return to work longer than is necessary for the protection of patients and coworkers around them, especially at a time when most facilities are recommending universal masking of HCP. For HCP who had relatively mildly symptomatic cases (ie, managed as an outpatient), a symptom-based strategy as recommended by the CDC appears appropriate based on the same data. For asymptomatic cases, questions remain about relative transmission risk and possibly about timing of infection related to testing. The CDC strategy of returning those HCP to work 10 days after their positive test seems reasonable. Staff who developed more severe COVID-19 infections (eg, those requiring hospitalization and/or critical care support) may represent a special case; as such, severely ill patients may shed virus for longer periods from symptom onset.^8,9^ In addition to the requirement that at least 10 days have passed since symptoms first appeared, the CDC guidelines also requires that at least 72 hours have passed since recovery, defined in their guidance as resolution of fever and improvement in respiratory symptoms.^1^ Adding the qualifier that “respiratory symptoms must have improved” to the “10 days from symptom onset” requirement may help address this issue. Additional, unanswered questions remain about how to manage immunocompromised HCP. Fewer data are available on viral viability duration in this latter population, and the need for further research on this topic is urgent.

Thus, based on these data, one approach to managing recovered HCP who work primarily with patient populations at high risk of complications would be to use the “time from symptom development” strategy plus “sustained improvement in respiratory symptoms” and add a safety factor (eg, adding an additional week or 2) to the time from symptom onset, or perhaps adding 2 weeks from test positivity for asymptomatic staff detected as positive. Institutions can decide how much of a cushion is appropriate, based on local factors, patient populations, etc. In addition, the use of source-control masking should be required for such individuals.

The dynamic nature of the pandemic necessitates that the healthcare epidemiology community continue to closely monitor the data as it emerges and to adjust policies and procedures, both to maximize patient and staff safety, while preserving the labor force that is essential to our ultimate success.
